# Implementation of Point-of-Care Diagnostics Leads to Variable Uptake of Syphilis, Anemia and CD4+ T-Cell Count Testing in Rural Maternal and Child Health Clinics

**DOI:** 10.1371/journal.pone.0135744

**Published:** 2015-08-26

**Authors:** Caroline De Schacht, Carlota Lucas, Nádia Sitoe, Rhoderick Machekano, Patrina Chongo, Marleen Temmerman, Ocean Tobaiwa, Laura Guay, Seble Kassaye, Ilesh V. Jani

**Affiliations:** 1 Elizabeth Glaser Pediatric AIDS Foundation, Maputo, Mozambique; 2 Instituto Nacional de Saúde, Maputo, Mozambique; 3 Elizabeth Glaser Pediatric AIDS Foundation, Washington DC, United States of America; 4 International Centre for Reproductive Health, Department of Obstetrics and Gynecology, Ghent University, Ghent, Belgium; 5 Clinton Health Access Initiative, Maputo, Mozambique; 6 Department of Epidemiology and Biostatistics, The George Washington University, Milken Institute School of Public Health, Washington DC, United States of America; Curtin University, AUSTRALIA

## Abstract

**Introduction:**

Anemia, syphilis and HIV are high burden diseases among pregnant women in sub-Saharan Africa. A quasi-experimental study was conducted in four health facilities in Southern Mozambique to evaluate the effect of point-of-care technologies for hemoglobin quantification, syphilis testing and CD4+ T-cell enumeration performed within maternal and child health services on testing and treatment coverage, and assessing acceptability by health workers.

**Methods:**

Demographic and testing data on women attending first antenatal care services were extracted from existing records, before (2011; n = 865) and after (2012; n = 808) introduction of point-of-care testing. Study outcomes per health facility were compared using z-tests (categorical variables) and Wilcoxon rank-sum test (continuous variables), while inverse variance weights were used to adjust for possible cluster effects in the pooled analysis. A structured acceptability-assessment interview was conducted with health workers before (n = 22) and after (n = 19).

**Results:**

After implementation of point-of-care testing, there was no significant change in uptake of overall hemoglobin screening (67.9% to 83.0%; p = 0.229), syphilis screening (80.8% to 87.0%; p = 0.282) and CD4+ T-cell testing (84.9% to 83.5%; p = 0.930). Initiation of antiretroviral therapy for treatment eligible women was similar in the weighted analysis before and after, with variability among the sites. Time from HIV diagnosis to treatment initiation decreased (median of 44 days to 17 days; p<0.0001). A generally good acceptability for point-of-care testing was seen among health workers.

**Conclusions:**

Point-of-care CD4+ T-cell enumeration resulted in a decreased time to initiation of antiretroviral therapy among treatment eligible women, without significant increase in testing coverage. Overall hemoglobin and syphilis screening increased. Despite the perception that point-of-care technologies increase access to health services, the variability in results indicate the potential for detrimental effects in some settings. Local context needs to be considered and services restructured to accommodate innovative technologies in order to improve service delivery to expectant mothers.

## Introduction

The reduction of maternal and infant mortality is dependent on the effective treatment of key diseases during antenatal care. The availability of timely laboratory diagnosis has been a long-standing challenge in resource-limited countries, especially in rural settings where maternal and child health (MCH) indicators remain poor. The arrival in the last decade of point-of-care (POC) technologies relevant to MCH services has brought renewed hope for increasing the coverage, breadth and quality of health care among women and children living in these territories. For the purpose of this study, POC testing referred to testing that was conducted at or near the patient, with no requirement for laboratory infrastructure, and that yielded results in a very short timeframe. These technologies can guide immediate treatment management decisions during the clinic visit [1]. For pregnant women, this may improve management of medical conditions associated with maternal and child morbidity and mortality. The recent introduction of POC testing for CD4+ cell counting in health systems [2–4] has stimulated a fresh debate about the quality and access to comprehensive diagnostics within MCH services.

Anemia is common among pregnant women in sub-Saharan Africa, aggravated by poor nutritional status, and parasitic infestation with organisms such as malaria and hookworm [5]. Worldwide, prevalence of anemia during pregnancy is estimated at 42% [6]. Congenital syphilis remains a major health problem worldwide and is often neglected [7, 8]. An estimated 1.5 million pregnant women are yearly infected with syphilis [9]. In addition, there is increased risk of vertical transmission of HIV among women co-infected with syphilis [10]. Screening for and treatment of syphilis during pregnancy can prevent congenital syphilis and decrease the risk of stillbirths [11]. In 2013, about 210,000 children in sub-Saharan Africa were newly infected with HIV, the majority through mother-to-child transmission (MTCT) [12]. Vertical transmission to infants is largely preventable with the use of antiretroviral medications. At the time of the study, the World Health Organization (WHO) guidelines recommended combination antiretroviral therapy (ART) for individuals with CD4+ T cell count less than 350 cells/μl or WHO stage III-IV, making CD4+ T cell enumeration necessary for eligibility determination. Providing ART in MCH or ensuring an immediate link from MCH to HIV services are crucial to avoid delays in treatment initiation as ART is a proven and effective MTCT prevention strategy [13].

In Mozambique pregnant women are highly burdened by HIV (15.8% prevalence, 2011[14]), syphilis (2.2% prevalence, 2011[14]) and anemia (52% prevalence, 2005 [6]). About 90% of the pregnant women attend antenatal clinics (ANC) at least once but only half attend all four visits, as recommended by WHO [15]. Comprehensive MCH services require access to screening and diagnostic assays to identify ailments and allow for timely interventions. Even when diagnostic tests are physically available within a health facility laboratory, pregnant women may not access these services if return visits to the facility are required for results and/or are associated with long waiting times. Since many years, testing for hemoglobin and syphilis for pregnant women have been available at health facilities’ laboratories in Mozambique, while samples for CD4+ cell enumeration among HIV-infected individuals are collected weekly and sent to a referral laboratory located within higher level health facilities.

The objectives of this operational research study were to determine: (i) the effect of the introduction of an integrated package of POC testing services for quantification of hemoglobin and CD4+ T cells, and syphilis serology within MCH services on coverage of testing; (ii) the effect of POC testing on initiation and timing of ART among ART-eligible HIV-positive pregnant women; and (iii) the acceptability of the use of POC technologies by health professionals.

## Methods

### Design, population, location

A quasi-experimental operational research study was conducted in four rural public health facilities in two provinces (Maputo and Gaza) in southern Mozambique. The sites (Moamba, Macia, Magude and Marracuene) were purposively selected due to the high volume of antenatal patients seeking MCH services and high prevalence of HIV, and for being health facilities supported by the Elizabeth Glaser Pediatric AIDS Foundation at the time of the study approval. Data were extracted from existing clinical charts and registers for two groups of women; the first group attended ANC before introduction of POC technologies for CD4+ T cell enumeration, hemoglobin and syphilis screening, and the second group after implementation of POC testing.

Before introduction of POC testing, hemoglobin and syphilis testing were performed at the laboratory associated with the health facility using Lovibond (Orbeco-Hellige, Florida, US) and Rapid Plasma Reagin (RPR) (Biotec Lab, Suffolk, UK), respectively. Laboratories at these health facilities possessed very basic infrastructure and equipment, were staffed by 3–4 professionals and had relatively inefficient linkages to the corresponding referral laboratory. Nurses at the antenatal consultation gave a test request to pregnant women, who then queued at the laboratory, located in a different part of the same health facility, for blood collection. Once blood was collected, pregnant women waited for their results at the laboratory. After results were returned to the pregnant women by laboratory staff, women went back to the antenatal consultation where they would wait until seen by the nurse. Although the result for hemoglobin and syphilis was available on the same day prior to this study, the inefficient flow was burdensome and time-consuming for the client. In addition, pregnant women had to provide a separate specimen for CD4 counting as described below.

Blood samples for CD4+ T cell enumeration were sent weekly to the referral laboratory, where testing was performed using the FACsCalibur (Becton Dickinson, San Jose, CA, USA). Nurses made an appointment for blood collection for CD4 counting on a fixed day, in general requiring therefore an extra visit. On the specimen collection day, women queued at the MCH clinic usually at very early hours of the day. The result on the CD4 counting would only be available on yet a different day, requiring an additional visit to the health facility. Antiretroviral therapy was initiated if CD4+ T-cell count was below 350cells/μL, the standard national policy at the time of the study. Initiation was done at the ANC clinic as soon as possible after determination of eligibility.

The POC technologies introduced in January 2012 were: 1) Hemocue 201+ (Hemocue AB, Angelholm, Sweden) for hemoglobin measurement; 2) Bioline 3.0 syphilis (Standard Diagnostics Inc., South-Korea) rapid test for syphilis diagnosis; and 3) Alere PIMA (Alere Inc., Waltham, Massachusetts, USA) for CD4+ T cell enumeration. All these technologies were placed within the MCH services. At each health facility, MCH staff, laboratory staff and the medical director of the health facility were trained on the study objectives and procedures. The change in patient flow due to the implementation of POC technologies was discussed and arranged with clinic staff input within the protocol training. Two nurses within each health facility were trained to perform the tests for routine care within their MCH services. In one HF (Macia), a clinical officer had been trained for CD4+ T-cell enumeration prior to the study and continued supporting that service. After the training, the three POC tests were exclusively performed by the MCH nurses at the clinic.

### Procedures

Women who attended their first ANC visit between January and December 2011 for the pre-POC evaluation, and between January and June 2012 for the post-POC evaluation, were eligible for the study. HIV-positive women were excluded from the analysis on coverage of CD4+ T-cell count enumeration and ART initiation if they were on ART at first ANC visit. The sample size for HIV-positive women was calculated separately for each of the health facilities based on a hypothesized 25% increase in ART uptake, using an 80% power and an adjustment for incompleteness of 5%. As data on uptake of hemoglobin and syphilis testing was limited at the time of protocol development, the study estimated to include an equal number of HIV-negative women. For the pre-POC period, files were randomly selected for review using systematic sampling. For the short post-POC evaluation, all files of the HIV-positive women were included in order to reach the desired sample size while for HIV-negative women files were randomly selected using systematic sampling.

Data were extracted from antenatal care cards, PMTCT register books and laboratory register books. HIV patient files were used to cross-check laboratory results and ART initiation. Test coverage was defined as a test result being available in either of 3 places: laboratory registry, clinic registry or antenatal cards. Time from HIV diagnosis and each step of the laboratory services up to ART initiation was measured: i) Diagnosis of HIV as written on the antenatal cards; ii) Registration of CD4 analysis request at the MCH clinic; iii) Registration of blood collection at the MCH clinic; iv) Registration of the analysis at the laboratory as written on the CD4 result printout; v) Registration of the results at the clinic as written on the PMTCT book; vi) Registration of results in patient file at the MCH clinic.

Structured interviews were conducted with MCH nurses and laboratory technicians to evaluate perceived and then actual acceptability of MCH nurses performing POC technologies, before and after introduction, respectively. The MCH nurses and laboratory technicians were informed about the study by the study team and interviews were conducted with those who provided consent.

### Data management and data analysis

Data were extracted onto data extraction forms and entered into a Microsoft Access database. Data were analyzed using STATA Version SE/11.1 (*Stata Corp*, *Texas*, *US*). Study outcomes were laboratory testing and treatment uptake for syphilis for all pregnant women, testing uptake for anemia for all pregnant women, uptake of immunological staging for HIV positive pregnant women and uptake of ART treatment for eligible women. As the study was a retrospective routine data review, being screened was defined as having a test result documented in the patient file. Eligibility for ART for the purpose of the study was defined as having a CD4+ T cell count below 351 cells/μL. Anemia is defined as having a hemoglobin <8mg/dl. For each health facility, we estimated the proportion with test before and after introduction of POC technologies and compared two proportions using z-tests. In the pooled analysis, we estimated the overall proportions before and after introduction by pooling across health facilities using an inverse-variance weighted estimate. The overall effect of the POC was estimated by pooling the health facility specific effects using inverse variance weights, a technique based on the random effects model used in meta-analysis. The random effects model accounts for potential intra-cluster (health facility) correlation in the estimation of robust standard errors.

### Ethics

The protocol was approved by the National Committee for Health Bioethics of the Ministry of Health, Mozambique and by the Ethics Committee of the George Washington University, Washington DC, US. Signed informed consent was obtained from all health worker interviewees. A waiver of signed consent was obtained from the Ethics Committees for performing chart reviews of participating pregnant women. Patient information was anonymized and de-identified prior to analysis.

## Results

**Fig 1:**
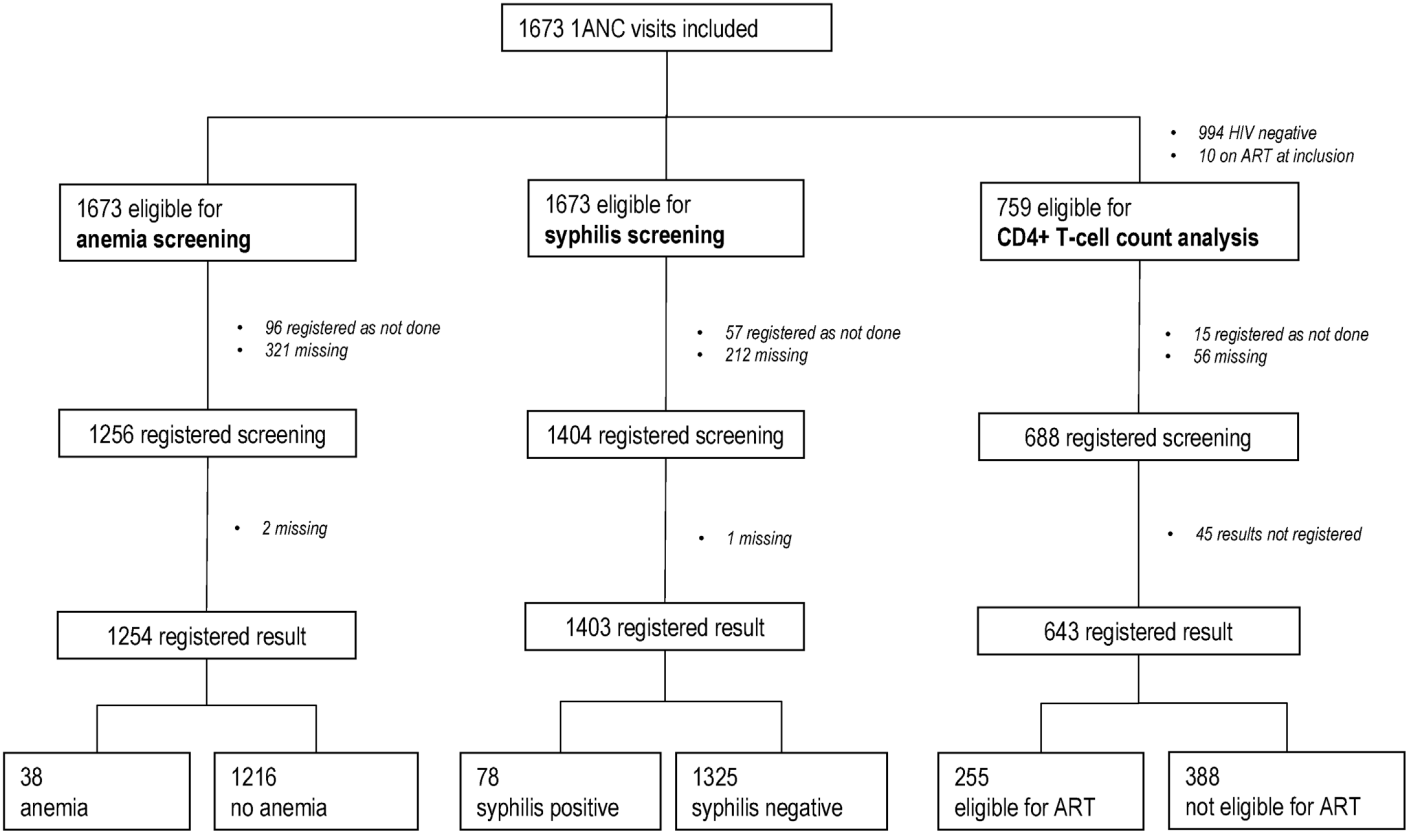
Consort Diagram. Flow of patient records’ review for screening of anemia, syphilis and analysis for CD4+ T-cell count and the results. Anemia is defined as a hemoglobin <8mg/dl. Eligibility for ART is defined as having a CD4+ T-cell count <351/mg/μL. ANC, antenatal care—ART, antiretroviral therapy.

A total of 1673 patient files were reviewed, 865 before introduction and 808 after introduction of the POC tests (Fig 1). Fig 1 describes the flow of patient record review. Anemia and syphilis screening was studied in 1673 eligible records of HIV-positive and HIV-negative women, while CD4 cell counting was investigated in files of 759 eligible HIV-positive women. Ten HIV-positive women were already on ART before the current pregnancy and excluded from the analysis for the CD4+ T-cell enumeration and ART initiation coverage.

Characteristics of the groups are shown in Table 1. There were no differences in the age and gestational age of women at first antenatal visit between the pre-POC and post-POC implementation periods. However, women in the post-POC implementation period had a lower median CD4 cell count.

**Table 1:** Characteristics of the study population (n = 1673).

	Pre-POC implementation	Post-POC implementation	p*
**Total (n, percentage)**	865	808	
Moamba	200 (23)	161 (20)	
Macia	203 (23)	224 (28)	
Magude	285 (33)	235 (29)	
Marracuene	177 (21)	188 (23)	
**Age at first antenatal care visit (years; median (Interquartile Range))**	24 (20–29) (n = 860)	24 (20–29) (n = 807)	0.42
**Gestational age at first antenatal care visit (weeks; median (Interquartile Range))**	23 (19–26) (n = 756)	22 (19–26) (n = 787)	0.05
**HIV positivity (n, percentage)**			<0.0001
HIV negative	413 (48%)	491 (61%)	
HIV positive	452 (52%)	317 (39%)	
**CD-4 T-cell count (HIV positive women only) (cells/μl (median, Interquartile Range)**	431 (285–622) (n = 388)	365 (230–526) (n = 255)	0.0003
**Eligibility for ART, defined as CD4+ T-cell count ≤350 cells/μL (HIV positive women only) (%)**	35	46	0.005

* Chi-square test for comparison of categorical variables; Wilcoxon test for continuous variables

Table 2 describes the characteristics of health facilities. In one health facility (Moamba), POC testing for CD4+ T-cell enumeration was available prior to this study and testing was performed by a medical officer serving patients with the highest need. With the initiation of the study, the device was moved to the MCH clinic and tests were performed by the MCH nurse, serving MCH services.

**Table 2:** Characteristics of the study health facilities.

Health Facility (Province)	Number of ANC attendees (2011)	% HIV-positive women in ANC (2011)	POC Technologies available before study	Number of MCH nurses trained on POC	Integration of ART services at MCH	Human Resource issues at MCH/HF
Macia (Gaza)	2744	25%	Yes—CD4+ T-cell enumeration	1	2008	2011: POC for CD4+ T-cell enumeration operated by medical officer at HF—used for hospitalized patients, pediatrics, pregnant women, referral from peripheral HF
						2012: operated by MCH nurse, serving MCH services
Moamba (Maputo)	755	14%	No	2	2013	Not reported
Magude (Maputo)	1525	21%	No	2	2013	Not reported
Marracuene (Maputo)	1355	21%	No	2	2013	1 nurse active as operator; 1 nurse only part-time as operator.
						POC CD4+ T-cell enumeration served patients from all sectors

POC—Point-of-Care; ANC—Antenatal Care; MCH—Maternal and child health; ART—Antiretroviral therapy; HF—Health Facility

### Coverage of screening tests

Table 3 highlights the results of the coverage of screening tests, before and after introduction of POC technologies.

**Table 3:** Coverage of registered screening tests among the study population.

***Coverage anemia screening***							
	Pre-POC implementation			Post-POC implementaton			
	Total	N	Coverage (%)– 95% CI	Total	N	Coverage (%)– 95% CI	p^1^
**Crude**	865	591	68.3 (65.2–71.4)	808	663	82.1 (79.4–84.7)	**<0.0001**
**Weighted** *	865	591	67.9 (45.3–90.6)	808	663	83.0 (74.5–91.4)	0.229
Moamba	200	128	64 (57.3–70.7)	161	151	93.8 (90.0–97.5)	**<0.0001**
Macia	203	71	35.0 (28.4–41.6)	224	173	77.2 (71.7–82.7)	**<0.0001**
Magude	285	230	80.7 (76.1–85.3)	235	189	80.7 (76.1–85.3)	0.937
Marracuene	177	162	91.5 (87.4–95.6)	188	150	79.8 (74.0–85.5)	**0.001**
***Coverage syphilis screening***							
	Pre-POC implementation			Post-POC implementaton			
	Total	N	Coverage (%)– 95% CI	Total	N	Coverage (%)– 95% CI	p^1^
**Crude**	865	712	82.3 (79.8–84.9)	808	691	85.5 (83.1–87.9)	0.075
**Weighted** *	865	712	80.8 (65.3–96.2)	808	691	87.0 (76.4–97.7)	0.282
Moamba	200	173	86.5 (81.7–91.3)	161	160	99.4 (98.2–100.6)	**<0.0001**
Macia	203	126	62.1 (55.4–68.8)	224	140	62.5 (56.1–68.9)	0.927
Magude	285	279	97.9 (96.2–99.6)	235	220	93.6 (90.5–96.8)	**0.014**
Marracuene	177	134	75.7 (69.4–82.0)	188	171	91.0 (86.8–95.1)	**<0.0001**
***Coverage CD4+ T-cell count screening***							
	Pre-POC implementation			Post-POC implementaton			
	Total	N	Coverage (%)	Total	N	Coverage (%)	p^1^
**Crude**	450	388	86.2 (83.0–89.4)	309	255	82.5 (78.3–86.7)	0.164
**Weighted** *	450	388	84.9 (74.7–95.2)	309	255	83.5 (69.0–98.0)	0.930
Moamba	100	78	78.0 (69.8–86.2)	50	42	84.0 (73.7–94.3)	0.386
Macia	133	129	97.0 (94.0–100)	87	51	58.6 (48.2–69.0)	**<0.0001**
Magude	139	126	90.6 (85.8–95.5)	91	82	90.1 (83.9–96.3)	0.842
Marracuene	78	55	70.5 (60.3–80.7)	81	80	98.8 (96.3–101.1)	**<0.0001**
***Time from HIV diagnosis (registered at fist ANC) to CD4+ T-cell count analysis date (registered at laboratory result) (days)***							
	Pre-POC implementation			Post-POC implementaton			
	N	median time (d)–IQR		N	median time (d)—IQR		p^2^
**Total**	350	9 (5–35)		245	0 (0–1)		**<0,0001**
Moamba	64	8 (3–56)		42	0 (0–1)		**<0,0001**
Macia	119	13 (4–35)		46	4 (0–30)		**0,004**
Magude	121	9 (6–35)		80	0 (0–0)		**<0,0001**
Marracuene	46	8 (7–22)		77	0 (0–0)		**<0,0001**
***Coverage all 3 POC tests—package (HIV positive women)*, *defined as result registered for 3 tests over HIV positive women attending 1st antenatal care visit***							
	Pre-POC implementation			Post-POC implementaton			
	Total	N	Coverage (%)– 95% CI	Total	N	Coverage (%)– 95% CI	p^1^
**Crude**	450	206	45.7 (41.2–50.4)	309	187	60.5 (54.9–65.8)	**<0.0001**
**Weighted** *	450	206	45.2 (10.7–79.6)	309	187	62.6 (25.9–99.3)	**0.158**
Moamba	100	65	65.0 (55.6–74.4)	50	39	78.0 (66.4–89.6)	0.104
Macia	133	13	9.8 (4.7–14.8)	87	12	13.8 (6.5–21.1)	0.358
Magude	139	103	74.1 (66.8–81.4)	91	66	72.5 (63.3–81.8)	0.792
Marracuene	78	25	32.1 (21.6–42.8)	81	70	86.4 (78.9–93.9)	**<0.0001**

IQR Interquartile Range; POC Point-of-Care

*Pooled differences estimates (%) with 95% CI: Coverage anemia screening 14.9 (-9.4–39.2); coverage syphilis screening 6.0 (-4.9–17.0); coverage CD4+ T-cell count -1.2 (-27.2–25.2); coverage all 3 POC tests 17.3 (-6.7–41.3)

^1^ z-test and logistic regression;

^2^ Wilcoxon rank sum test

#### Hemoglobin testing

Weighted pooled analysis showed an increase, although not significant, in testing coverage from 67.9% (95%CI: 45.3–90.6) to 83.0% (95%CI: 74.5–91.4) (p = 0.229). The pooled weighted difference estimate was 14.9% (95% CI: -9.4–39.2). Per health facility (HF) analyses showed significant increases in testing coverage in two HF with initial low coverage (Moamba and Macia) but significantly lower testing in the one HF that had high coverage initially (Marracuene). Hemoglobin testing uptake was not significantly higher among HIV negative women compared with HIV-positive women prior to POC testing (77.2% vs. 60.2% respectively; p = 0.47), with higher, only marginally different results after introduction of POC testing (84.1% vs. 78.9%; p = 0.06). There was no significant difference in time from first ANC visit to hemoglobin screening between pre- (median 1 day) and post- POC (median 1 day). Severe anemia (defined as Hb <8 mg/dl) was twice as high in HIV positive compared with HIV negative women in the combined pre-and post-cohort (OR 1.96, p = 0.04), but anemia treatment and clinical responses to these data were not available for abstraction.

#### Syphilis testing

Coverage of syphilis screening was high initially and did not change significantly after introduction of the POC testing (80.8% (95%CI: 65.3–96.2) vs. 87.0% (95%CI: 76.4–97.7); p = 0.282), with a pooled weighted difference of 6.0% (95% CI: -4.9–17.0). There was no difference in testing uptake by HIV status. The effect of introduction of POC technologies on syphilis screening varied across the four health facilities. Syphilis screening rates significantly increased in Moamba and Marracuene, were low initially and remained unchanged in Macia, and significantly decreased in Magude. Analysis of time from first ANC visit to syphilis screening did not show any difference, with same-day testing being performed both before and after introduction of POC testing.

Overall rate of syphilis positivity was three times higher among HIV positive pregnant women compared to HIV negative women (OR 0.34; p<0.0001). With the use of the non-treponemal tests in the pre-POC period, syphilis positivity was 5.1% and 12.0% among HIV-negative and HIV-positive women, respectively. With the use of the more specific treponemal POC test, positivity rates dropped to 1.5% and 5.5% among HIV-negative and HIV-positive women, respectively. Initiation rate of syphilis treatment did not differ before [56/58 (96.6%)] and after introduction of POC testing [19/20 (95.0%)], although with POC testing women were more likely to be correctly identified as having syphilis.

#### CD4+ T cell count analyses

The weighted pooled analysis did not show a significant difference in CD4+ T cell count testing among women identified at ANC with HIV (84.9% (95%CI: 74.7–95.2%) vs. 83.5% (95%CI: 69.0–98.0%); p = 0.930). However, health facility analyses showed a significant increase in CD4+ T cell count testing in Marracuene (70.5% to 98.8%) but a significant decrease in Macia, the facility with POC testing available prior to the study (97.0% to 58.6%). The median time from identification of HIV at ANC to CD4+ T cell count testing decreased significantly in the weighted pooled and stratified analysis.

#### Coverage of the package of 3 screening tests

More women had all three screening tests performed when using POC testing within MCH. No significant difference was seen in the weighted pooled analysis and stratified analysis, except for one health facility with a significantly higher uptake (32.1% vs 86.4% before vs after introduction) in Marracuene. The very low coverage in Macia pre-POC of 10% is explained by very low hemoglobin screening among HIV positive women (11%). The post-POC coverage in the same facility increased but remained low mainly due to the low CD4 T cell testing uptake.

### Coverage of ART initiation

**Fig 2:**
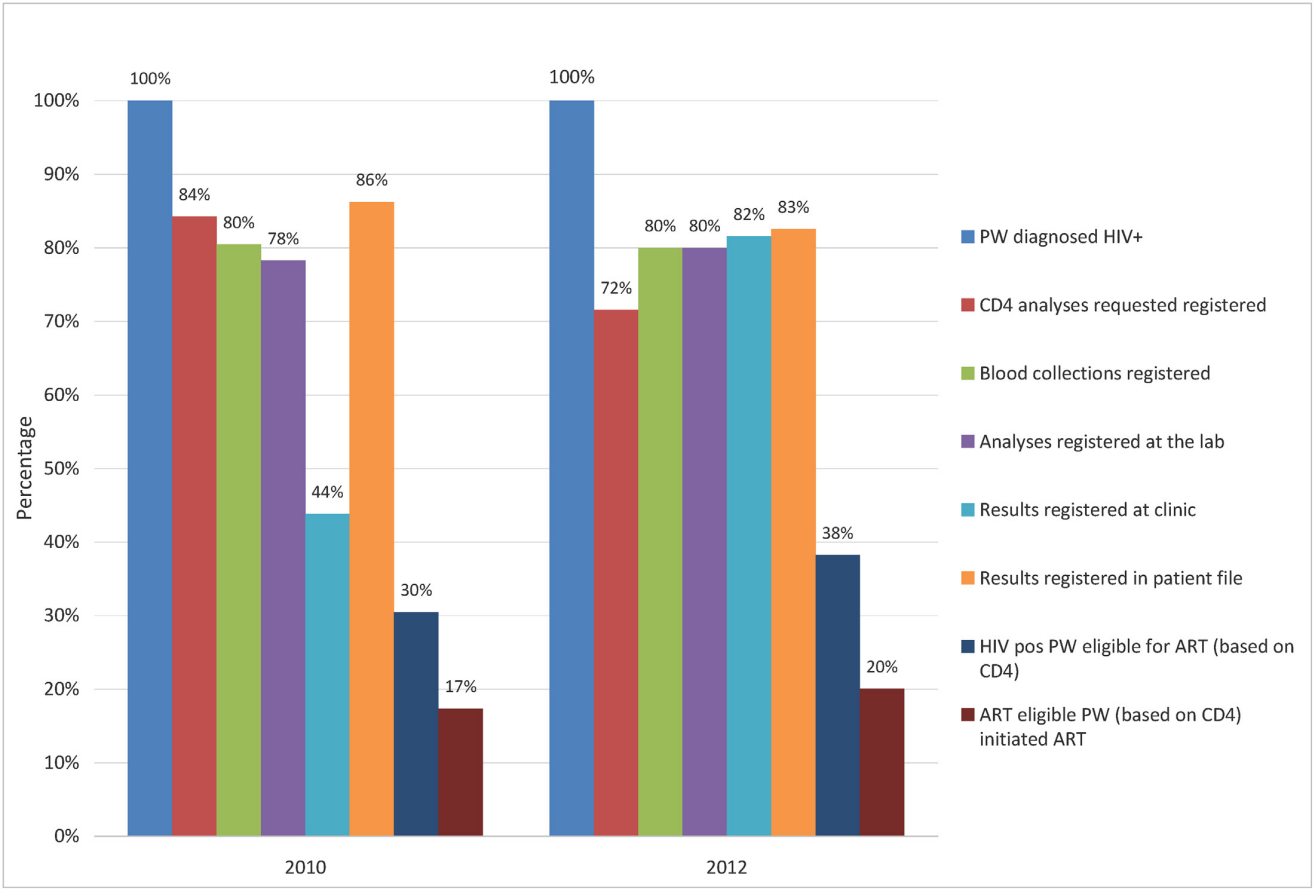
Cascade HIV/ART services for HIV positive pregnant women, pre-and post-introduction of POC CD4+ T-cell count. Cascade HIV/ART services for HIV positive pregnant women, from time of registration of HIV status to ART initiation, pre-(2010) and post-(2012) introduction of the point-of-care testing for CD4+ T-cell enumeration performed at the maternal and child health services. All proportions are calculated with the denominator being the number of women diagnosed with HIV. Eligibility for ART is defined as CD4+ T-cell count <351cells/μL. PW—Pregnant Women; ART—Antiretroviral Treatment.

Eligibility for ART initiation based on CD4+ T cell count below 351 cells/μL was 35% in 2011 versus 46% in 2012. The cascade of HIV positive women from diagnosis to ART initiation is shown in Fig 2. In the pre-POC period, there was a large drop-off in the number of CD4 test results that were documented at the HF, which was not present in the post-POC period.

Initiation of ART among eligible women was similar in the weighted pooled analysis (pooled weighted difference of -3.7% (95% CI: -54.0–46.7); p = 0.887) but significant differences were observed in two HF (increase in Macia (40.9% to 91.3%) and decrease in Marracuene (85.7% to 22.9%)) (Table 4). The time from HIV diagnosis to ART initiation showed a trend of decrease from a median of 50 days initially versus 16 days post-POC testing. In the HF analysis, time decreased in all study sites and reached statistical significance in three of them (Table 4).

**Table 4:** Coverage of ART initiation for ART-eligible women, registered at the health facility.

***Coverage ART initiation for ART-eligible women***							
	Pre-POC implementation			Pre-POC implementation			
	# eligible	# initiated	Coverage (%)– 95% CI	# eligible	# initiated	Coverage (%)	p ^1^
**Crude**	137	78	56.9 (48.5–65.0)	118	62	52.5 (43.5–61.5)	0.48
**Weighted** *	137	78	61.4 (42.1–80.7)	118	62	57.9 (25.0–90.9)	0.887
Moamba	22	14	63.6 (41.7–81.0)	22	16	72.7 (50.4–87.5)	0.52
Macia	44	18	40.9 (27.3–56.0)	23	21	91.3 (70.3–97.9)	**<0.0001**
Magude	50	28	56.0 (41.9–69.2)	38	17	44.7 (29.7–60.8)	0.3
Marracuene	21	18	85.7 (11.7–39.8)	35	8	22.9 (11.7–39.8)	**<0.0001**
***Time from HIV diagnosis to ART initiation for eligibles (days)***							
	Pre-POC implementation			Pre-POC implementation			
	N	median time (d)–IQR		N	median time (d)—IQR		p ^2^
**Total**	78	44 (31–95)		62	17 (6–38)		**<0,0001**
Moamba	14	62 (31–102)		16	6 (3–17)		**0.0002**
Macia	18	54 (33–103)		21	24 (8–40)		**0.008**
Magude	28	40 (24–77)		17	34 (29–69)		0.61
Marracuene	18	41 (32–65)		8	12 (7–22)		**0.002**

ART—Antiretroviral Therapy; POC—Point-of-Care; IQR—Interquartile Range; CI- Confidence Interval

*Pooled differences estimates with 95% CI: -3.7 (-54.0–46.7)

^1^z- test and logistic regression;

^2^Wilcoxon rank sum test

### Acceptability

A survey was conducted among MCH nurses and laboratory technicians to assess acceptability of performing POC at MCH. Results were similar in the pre- and post-evaluation for most questions (Table 5). Although in the pre-evaluation participants thought that workload would be high when using POC at MCH, this was not reported as an ongoing concern after the introduction of the tests (86% before versus 21% after; p = 0.001). Most health workers preferred POC testing to laboratory-based testing, both before and after the implementation of POC assays in their health facilities. Although more participants were confident that more women would be initiating ART when eligible, both in the pre-and post-evaluation, a lower percentage felt this after introduction (77% before versus 59% after; p = 0.05).

**Table 5:** Acceptability of POC tests performed at MCH by MCH nurses (n = 33) and laboratory technicians (n = 8), perceived pre-POC and actual post-POC implementation at MCH.

	Pre-POC implementation (n = 22)		Post-POC implementaton (n = 19)		P^1^
Age					
Median (IQR)	30 (27–38)		29 (25–37)		
Missing	1		1		
	n	%	n	%	
Sex					0.56
Male (n, %)	18	82	15	80	
Missing	4	18	4	20	
Do you think performing POC tests is easy?					0.25
Very easy	5	23	1	5	
Easy	14	64	15	79	
Difficult	2	9	0	0	
No opinion	1	4	2	11	
Missing	0	0	1	5	
Will POC tests done at MCH services have problems?					0.27
No mistakes	19	86	14	74	
Some problems might happen	3	14	5	26	
Lots of problems	0	0	0	0	
Will POC tests give comparable results at MCH services as with results from the laboratory?					0.65
Comparable results	19	86	14	74	
Some mistakes	3	14	2	11	
Missing	0	0	3	16	
Will health care workers be able to attend more women with POC tests performed at MCH?^2^					0.59
Strongly agree	1	5	2	11	
Agree	11	50	9	47	
Disagree	4	18	1	5	
No opinion	2	9	3	16	
Missing	4	18	4	21	
Will more women initiate antiretroviral treatment when POC tests done at MCH?^2^					**0.05**
Same or less	1	5	0	0	
More	17	77	11	59	
Maybe	0	0	1	5	
No opinion	0	0	3	15	
Missing	4	18	4	21	
Will you have more work than before POC testing?^2^					0.45
Same	2	9	1	5	
More work	13	59	10	53	
Less work	3	14	1	5	
Initially more, but later the same	0	0	0	0	
No opinion	0	0	2	11	
Missing	4	18	5	26	
Which do you prefer?^2^					0.55
POC testing	17	77	15	79	
Routine	1	5	0	0	
No opinion	0	0	0	0	
Missing	4	18	4	21	
How will workload be during one single consultation? ^2^					**0.001**
Low	3	14	10	53	
High	19	86	4	21	
Missing	0	0	5	26	

*POC- Point-of-Care; IQR—Interquartile Range; MCH—maternal and child health*

^1^ Fisher Exact’s Chi square test;

^2^Questions asked only to MCH nurses

## Discussion

Our study shows that POC tests performed at MCH services within primary health care facilities are potentially beneficial and well accepted by health professionals. The registration of diagnostics results dramatically improved with the implementation of POC diagnostics, increasing, therefore, the likelihood of clinical action based on testing results. Additionally, the implementation of an integrated package of three POC assays led to an overall increase of the coverage of all three, hemoglobin, syphilis and CD4+ cells tests, among pregnant women. Nevertheless, the impact on the coverage of all three assays or on the coverage of single tests was variable, probably due to differences in conditional factors [16]. Generally speaking, the impact was more significant in health facilities where the baseline coverage was low. Of note, in some health facilities a significant decline in testing coverage was seen after the introduction of new technologies. This was mainly observed in health facilities with initial high coverage of testing and is probably due to the disruption of existing efficient work processes.

The adoption of new health technologies in health systems must be well deliberated, carefully planned and well monitored to avoid negative consequences on the quality of care provided by health facilities [1, 16, 17]. Task shifting of some necessary screening tests to trained lay counselors should be evaluated. Several studies among non-pregnant women showed that performance of CD4+ POC by non-laboratory technicians produces reliable results [2, 18]. Availability of multiplex instruments in which multiple tests are performed using a single finger prick may further improve work flow and increase the feasibility of conducting multiple tests closer to patients without substantial increase in work burden [19]. The impact on service delivery of any of these approaches would need to be monitored to ensure that there are no unintended consequences to such changes.

The decision about the implementation of new diagnostic technologies in health systems is based on several complex and interplaying factors [16, 20, 21]. The cost and cost-effectiveness of the assays are critical considerations. Recent studies in resource-constrained settings have shown that POC syphilis testing and POC CD4+ cell counting are very cost-effective [20, 22]. Another important issue is if the novel technology has an impact on patient-important outcomes, such as morbidity, mortality or quality of life [3, 20]. Hemoglobin screening increased significantly when test was offered at the MCH clinic. Since anemia has high disease burden for pregnant women and their infants in resource-limited settings [6], having quantitative rapid tests at POC can make a substantial difference in their health status. Syphilis screening has been successfully implemented into MCH services, even before the availability of POC testing: our data confirms coverage of about 83%, almost reaching the WHO target of 90% [21]. Although time to hemoglobin and syphilis result did not change in this study, the higher accuracy of POC assays we introduced is most probably associated with better diagnosis and more appropriate clinical management. For example, after introduction of the syphilis POC treponemal test the positivity rate fell from 8.1% to 2.9% due to the better specificity of the assay [23, 24], reducing thus the number of women receiving unnecessary treatment for syphilis. Surprisingly, no significant increase in testing uptake was seen when syphilis diagnosis was offered at MCH services, in contrast to the results of a study in Uganda and Zambia [25]. Results for CD4+ T cell count testing and ART initiation for eligible women showed variability across study sites.

One of the most significant results of this study is that time to ART initiation decreased from 44 to 17 days with the use of POC diagnostics, reducing the probability of vertical transmission of HIV due to a faster drop in the viral load. Additionally, the proportion of eligible women initiating ART increased significantly in those health facilities where initial coverage was low. Interestingly, the absolute decrease in the time to ART (27 days) was higher than the one observed for CD4+ T-cell results (9 days), indicating that other factors may be at play once POC diagnostics are introduced. These could include more streamlined and efficient processes within the health facilities, better predictability of test results leading to more client satisfaction and less missed clinical appointments, and more responsiveness from health workers due to added empowerment from POC diagnostic services [16]. In one of the facilities (Macia), where a pre-existing POC was located outside MCH services, we saw a decrease in coverage of CD4+ T-cell screening among HIV positive pregnant women after changing the location of the device to within MCH services. This is probably due to a disruption of a pre-existing process that worked efficiently. At the same time, the proportion of women starting ART increased in this site as women were identified as eligible at the clinic, with less being lost to follow-up before ART initiation. On the other hand, in Marracuene health facility, more women got their CD4+ T-cell enumeration done, but less eligible women started treatment. When evaluating the process here, we found that the MCH nurse was performing the test for non-pregnant women, taking away her time for preparing and initiating treatment of HIV positive pregnant eligible women. In all of the four health facilities, there was no change in number of nurses after introduction of POC technologies. As countries in sub-Saharan Africa implement the Option B+ for PMTCT more widely in the coming years, the importance of POC testing may have less impact on the number of pregnant women that initiate ART [26]. However, immunological screening will still be necessary for optimal clinical management and monitoring of adherence where viral load is not available. Therefore, having POC devices at the clinic may remain essential.

To our knowledge this is the first report on the acceptability by health workers of a package of point-of-care diagnostic services aimed at routine ANC in sub-Saharan Africa. Our results show that POC testing was the preferred option by health professionals both before and after the introduction of these diagnostic tools in the routine work of the health facility, and that there was a shift in the perception of workload from high to low once the new routine was adopted. However, with the introduction of POC diagnostics there was less optimism among health workers regarding the impact of these technologies on the number of women initiating ART, probably due to the realization that diagnostic delays were not the sole factor influencing quality of care and that multiple complex factors were at play [17, 27]. The high acceptability of POC assays may also be a reflex of the current weakness of laboratory systems throughout sub-Saharan Africa [16] and of the empowering nature of these novel tools [16, 28].

Our study had several limitations: our retrospective chart review revealed missing data. As it was impossible to know if missing data were data not registered or tests not done, we only considered registered information in our calculations of screening coverages. This also potentially influenced the eligibility data we found in our study since we found that more women in the post-POC implementation period had lower CD4+ T-cell counts and were ART eligible. There has been a trend for higher CD4+ T-cell counts at ART initiation in Mozambique, with an increase of around 10 cells/mm^3^ per year [29]. Nevertheless, CD4+ T-cell counts at ART initiation continue to be well below 350 cells/mm^3^ since patients usually present late for care [30]. There is no specific data for CD4+ T-cell count trends among pregnant women in Mozambique. The difference we observed between the two periods could be caused by seasonal variations of CD4+ T-cell counts, changes in the influx of patients as a consequence to the introduction of a new POC assay or artifacts due to missing data. We also do not know if some women went to another HF for care, which could result in our underestimating the appropriate follow-up and use of test results for management. Other factors such as support systems in the HF, supervision, and monitoring can influence implementation of POC testing, and these were not measured or accounted for in our study.

## Conclusion

This study reveals that while POC tests for hemoglobin, syphilis, and CD4+ T-cell counts can greatly improve the delivery of ANC services, having access to these technologies does not mean that this potential is realized. There was significant variability in testing coverage before and after implementation of POC diagnostics across the four study sites and across the three tests performed. Multiple factors (such as site characteristics, patient volume, patient flow, staffing) may significantly impact the implementation of POC testing services. In general, POC testing performed at MCH is well accepted by health workers. Although there is a shift in perceived decreased workload after its introduction, there is no perception of a potential positive impact on initiation of ART. Further research is needed to identify optimal health delivery strategies to effectively bring the impact of technological advances to patients that are most at need.
